# Adapting to the Pandemic: Protocol of a Web-Based Perinatal Health Study to Improve Maternal and Infant Outcomes

**DOI:** 10.2196/30367

**Published:** 2021-09-10

**Authors:** Golfo Tzilos Wernette, Kristina Countryman, Okeoma Mmeje, Quyen M Ngo, Caron Zlotnick

**Affiliations:** 1 Department of Family Medicine University of Michigan Medical School Ann Arbor, MI United States; 2 Department of Obstetrics and Gynecology University of Michigan Medical School Ann Arbor, MI United States; 3 Department of Health Behavior and Health Education University of Michigan School of Public Health Ann Arbor, MI United States; 4 Butler Center for Research Hazelden Betty Ford Foundation Center City, MN United States; 5 Department of Psychiatry and Human Behavior Warren Alpert Medical School of Brown University Providence, RI United States; 6 Department of Medicine Women and Infants Hospital Providence, RI United States; 7 Department of Psychiatry and Mental Health University of Cape Town Cape Town South Africa

**Keywords:** COVID-19, pregnancy, COVID-19 pandemic, alcohol use, drug use, sexually transmitted infections, technology-delivered interventions

## Abstract

**Background:**

The identification of interconnected health risks during the perinatal period offers an opportunity to prevent negative maternal and infant health outcomes. Marijuana, opioid, and other substance use during pregnancy is a rapidly growing public health concern with significant and costly health consequences for the woman and the developing fetus. Pregnant persons who misuse substances are disproportionately more likely to engage in risky sexual behaviors resulting in sexually transmitted infections (STIs), which are on the rise in this population and can lead to adverse effects on maternal health and on fetal development.

**Objective:**

Our goal is to continue testing an innovative and low-cost technology-delivered intervention, the Health Check-Up for Expectant Moms (HCEM), which simultaneously targets alcohol and drug use and STI risk during pregnancy, both of which are on the rise during the COVID-19 pandemic.

**Methods:**

We describe the ways in which we have adapted the web-based HCEM intervention to continue recruitment and study enrollment during the pandemic.

**Results:**

Study recruitment, visits, and participant safety assessments were all successfully modified during the initial year of the COVID-19 pandemic. Compared to in-person recruitment that occurred prepandemic, remote recruitment yielded a greater proportion of women enrolled in the study (83/136, 61.0% vs 43/52, 83%) in a shorter period (12 months vs 7 months).

**Conclusions:**

Despite study challenges related to the pandemic, including time and effort adapting to a remote protocol, remote recruitment and visits for this study were found to constitute a successful approach.

**Trial Registration:**

ClinicalTrials.gov NCT03826342; https://clinicaltrials.gov/ct2/show/NCT03826342

**International Registered Report Identifier (IRRID):**

DERR1-10.2196/30367

## Introduction

### Overview

Alcohol, marijuana, opioid, and other substance use during pregnancy is a rapidly growing public health concern with significant and costly health consequences for the woman and developing fetus [1]. Women who misuse substances are disproportionately more likely to engage in risky sexual behaviors that can result in sexually transmitted infections (STIs). Pregnant persons are a scientifically complex group as national STI prevalence rates are on the rise among this population, leading to adverse effects on maternal health and on fetal development [2].

### COVID-19 and Women’s Health

The impact of the COVID-19 pandemic on women’s health has been significant. Women have reported experiencing more severe stress than men resulting in greater health impact, and pregnant women have reported more health-related worry and high levels of anxiety directly related to the COVID-19 pandemic [3,4]. Pregnant women are particularly impacted by the pandemic as they are at increased risk for severe illness compared to nonpregnant women, and they may be at higher risk for preterm birth [5]. Since the onset of the COVID-19 pandemic, there are numerous reports that women’s alcohol and other drug use has been rising in the United States. The US Centers for Disease Control and Prevention reported that approximately 12% of adult women reported either beginning or increasing their substance use to cope with pandemic-related stress [6]. Frequency of binge drinking—for women, defined as four or more drinks on one occasion—has increased substantially (over 40%) during this time, and cases of alcohol-related liver diseases have increased, especially among young women [7]. Moreover, marijuana use continues to escalate among pregnant women, with the most commonly cited reasons for use cited as relief of stress or anxiety, nausea or vomiting, and pain [8]. Pregnant women with opioid use disorders have faced unique challenges to care during the pandemic due to their complex health care needs (eg, clinic travel to receive medication and stigma) [9].

The co-occurrence of alcohol and substance use and sexual risk-taking contribute significantly to STI acquisition. With respect to the impact of the COVID-19 pandemic on sexual health, STIs were already at record highs before the pandemic and climbing, especially for childbearing women. Recent reports (2019) reveal increases from the previous year in the prevalence of gonorrhea, chlamydia, and syphilis of 5%, 3%, and 14%, respectively; among women of childbearing age, there was a 36% increase in syphilis cases [2]. Furthermore, there was a 40% increase in congenital syphilis cases, and an alarming 22% increase in newborn deaths related to congenital syphilis during the same time period [2]. Access to STI screening and treatment has been limited during the pandemic due to restrictions [10], likely leading to continued health consequences among this group.

The perinatal period has been identified as an urgent time to address and prevent these co-occurring risks [11], and technology-delivered interventions are ideally suited given their low cost and potential reach [12]. We are currently testing an innovative and low-cost technology-delivered intervention, the Health Check-Up for Expectant Moms (HCEM) (see study protocol in Tzilos Wernette et al [13]), which is theoretically grounded, consistent with motivational interviewing, and informed by the Information-Motivation-Behavior model, simultaneously targeting alcohol and drug use risk and risky sexual behavior during pregnancy. The HCEM is a 60-minute intervention that is guided by a narrator; provides information, including short video testimonials, highlighting the bidirectional relationships among these risk factors; and provides motivational strategies to enhance behavioral skills, including male and female condom use. In this paper, we highlight the ways in which our study team has adapted our research protocol and study procedures so that we can continue recruitment, assessment, and intervention remotely during the COVID-19 pandemic. Remote study participation may have potential advantages for participants, including lessening the burden of travel time, costs, and inconvenience associated with in-person visits [14].

## Methods

Our study uses a two-group, randomized controlled design with a baseline session (prior to 22 weeks pregnant), plus two brief booster sessions within 1 month of study enrollment. We conduct three follow-up assessments at 2 and 6 months from baseline, and a postpartum assessment at 6 weeks postpartum. All sessions are asynchronously delivered via technology using the Computerized Intervention Authoring Software [15]. Additionally, each session includes the assessment of risk behaviors (eg, risky sexual behavior and alcohol and drug use) using the “timeline follow-back” interview method, a calendar-assisted structured interview [16]. Study recruitment prior to the pandemic was conducted exclusively in person at obstetric and primary care clinics. During the pandemic, recruitment efforts shifted to remote (eg, phone, text messaging, and online). Study participants include 250 pregnant women, aged 18 years or older; participants endorse the following risk factors in order to be eligible for the study: (1) unprotected sex in the past 30 days in addition to having more than one male partner in the last 6 months and/or having uncertainty about current sexual partner’s monogamy and (2) current alcohol and drug use risk [17,18]. The study protocol was approved by the University of Michigan Medical Institutional Review Board (HUM00143896) and registered at ClinicalTrials.gov (NCT03826342).

## Results

### Overview

All in-person behavioral research studies university-wide were paused in March 2020 due to the COVID-19 pandemic. This impacted our study in three important ways. Primarily, we could no longer recruit new participants or conduct any study visits, including assessments and viewing of the intervention or control conditions. Next, STI testing and biological (eg, hair and urine) sample collection for the assessment of drug use was suspended since this was no longer done in person during a perinatal study visit. Furthermore, because we were no longer seeing women in person, we had to modify our guidelines around assessing for participant safety, including the remote assessment of suicidality. Finally, the following modifications to our technology-based intervention and protocol were made in order to continue during the pandemic. The changes we implemented are detailed below in Table 1 [19], by category; for each point, we describe the original protocol followed by modifications (ie, “current protocol”).

**Table 1:** Modifications to the Health Check-Up for Expectant Moms protocol in response to the COVID-19 pandemic.

Study aspects and protocols			Details
**Study visits**			
	**Baseline visits**		
		Original protocol	Baseline visits were conducted in person with research staff via interviews and accessing the CIAS^a^ platform using a study iPad.
		Current protocol	Baseline visits were adapted to be completed over the telephone and online through an individualized weblink to the CIAS platform.
	**Delivery of follow-up assessments**		
		Original protocol	Delivery of follow-up assessments (2 and 6 months from baseline and 6 weeks postpartum) was conducted in person with research staff via interviews and accessing the CIAS platform using a study iPad.
		Current protocol	Delivery of all follow-up assessments was modified to be completed over the phone and through an individualized weblink to the CIAS platform.
	**Collection of biological samples**		
		Original protocol	Collection of biological samples (urine and hair) for drug use assessment was conducted in person at clinic by research staff.
		Current protocol	Collection of biological samples is temporarily suspended.
	**Incentive payments**		
		Original protocol	Incentive payments (US $200 in cash) were made in person at study visits.
		Current protocol	Incentive payments (US $200) are made via electronic gift card (emailed) or check (mailed).
	**Modified COVID-19 assessment^b^**		
		Current protocol	A modified COVID-19 assessment was added to every study visit: the COVID-19 Family Stress Screener [19] was adapted for our study.
	**Intervention arm content**		
		Original protocol	Intervention arm content included references to booklet given in person and to the male and female condom demonstrators, which was part of the in-person intervention.
		Current protocol	Intervention arm content was updated to reflect remote nature of visits (eg, no longer refers to booklet given in person or to male and female condom demonstrators).
	**Informed consent**		
		Original protocol	Signed informed consent was obtained by research staff in person at the clinic.
		Current protocol	Informed consent process is conducted over the phone (copy of consent emailed to participants) and the research staff obtains consent verbally.
	**Safe sex kits**		
		Original protocol	Safe sex kits (eg, male and female condoms and dental dams) and study booklets were offered to the participants in person during the intervention study visit.
		Current protocol	Safe sex kits and study booklets are mailed to interested intervention participants with their permission.
	**Informational handouts**		
		Original protocol	All informational handouts (intervention and control) and local resources were provided to the participants in person at study visits.
		Current protocol	All informational handouts and list of local resources are emailed, including the study booklet for participants in the intervention arm.
	**Provider-ordered sexually transmitted infection (STI) tests**		
		Original protocol	STI test results (urine samples) were collected at our clinic labs at each study visit.
		Current protocol	New data are collected of all provider-ordered STI tests during participant’s pregnancy and postpartum period (not part of original protocol, in which we only collected study STI test results).
**Recruitment**			
	**Potential participants**		
		Original protocol	Potential participants were identified from clinic schedules and approached by research staff in person at clinics.
		Current protocol	Potential participants are still identified from clinic schedules, but recruitment resumes remotely via telephone calls, text messages, and email messages.
	**Call patients^b^**		
		Current protocol	Patients are not called until after their second obstetrics visit to help ensure active pregnancy (modified during COVID-19 pandemic, given remote procedures).
	**Interested patients**		
		Original protocol	Interested patients met with research staff to complete the screening survey in person at the clinic using the study iPad.
		Current protocol	Interested patients are emailed a weblink to complete the screening survey online.
	**Screening consent form**		
		Original protocol	The screening consent form was offered to the patient in person prior to completing the survey questions.
		Current protocol	The screening consent form appears on the webpage before any survey questions can be viewed.
	**Compensation**		
		Original protocol	All prospective study participants (eligible and ineligible) were physically given a US $5 gift card for their time by the research staff upon completing the screening survey at the clinic.
		Current protocol	All prospective study participants are emailed US $5 electronic gift card for their time upon completing the screening survey.
	**Eligible patients**		
		Original protocol	Eligible patients were scheduled for the baseline visit and consented in person at the clinic.
		Current protocol	Eligible patients are contacted by the research assistant via telephone, where more detailed study information is given; a copy of the consent form is emailed; and a baseline visit is scheduled for interested patients.
**Participant safety, suicide risk assessment, and child abuse and neglect**			
	**Modified procedures**		
		Original protocol	We did not include questions explicitly asking about suicide risk or child abuse and neglect. Participants that spontaneously indicated any distress from the study were given a list of referral options that included information on how to contact a social worker within our health system and/or were assessed by clinic social worker in person during the study visit.
		Current protocol	We modified our procedures for suicide risk assessment to reflect remote nature of visits (ie, research staff can no longer go to clinic social worker with participant to assess severity in person, as was done in the original protocol). We also modified our protocol for steps to take if our study team becomes aware of child abuse and neglect remotely, including procedures for how to report.
	**List of local resources**		
		Original protocol	A list of local resources was provided to all participants at the baseline study visit.
		Current protocol	An updated list of local resources is emailed and includes online resources (eg, Alcoholics Anonymous and Narcotics Anonymous remote meetings) during COVID-19 and changes to hours and/or procedures for all other local resources (eg, food pantries, counseling, and STI testing).

^a^CIAS: Computerized Intervention Authoring Software.

^b^This study aspect only came about as a result of the COVID-19 pandemic and only includes a current protocol.

### Participant Recruitment

While the pandemic caused significant initial delays to our study recruitment, since adapting to remote recruitment, we have exceeded the total number of patients screened compared to prepandemic recruitment. Remote recruitment over a 7-month period, from August 2020 to March 2021, has yielded 1122 contacts, with 305 women being screened. In the previous 12 months, from April 2019 to March 2020, we approached 892 women in the clinics and screened 232 women. Furthermore, of all women who were screened and were eligible prior to the pandemic, only 61.0% (83/136) went on to enroll in the study, whereas during remote recruitment, a greater proportion (43/52, 83%) of women enrolled in the study.

## Discussion

### Principal Findings

In this paper, we provide an overview of the HCEM intervention and study methodology. The COVID-19 pandemic has been disruptive to most facets of life globally, including research efforts. We highlight the ways in which we successfully adapted our study protocol to continue recruitment and intervention efforts with pregnant women during the pandemic. Study recruitment, visits, and participant safety assessments were all modified during the initial year of the COVID-19 pandemic.

Despite the challenges of the pandemic on women’s health and intervention efforts, there have been silver linings. Clinically, reduced health care visits and more telehealth visits have had some positive impacts on pregnant women [20-22]. Across the country, clinical treatment programs for substance use have ramped up telehealth utilization, offering care through a mix of telephone and/or video visits, with limited in-person visits to reduce COVID-19 exposure risk. This has been particularly beneficial to perinatal women seeking care for substance use disorder, including opioid use disorder, with relaxed regulations around travel to crowded clinics to receive treatment medications (eg, methadone) and psychotherapy, which help to overcome known treatment barriers (ie, stigma, health care access, transportation, and childcare) in this population [9,20-22]. Our own research has also seen positive impacts as a result of the COVID-19 pandemic, particularly in terms of study recruitment. The ability to offer “contact-free” visits alleviates anxiety surrounding the risks of in-person visits, and the convenience of being able to take the study screener and assessments from home seems to have increased interest in study participation.

Remote recruitment yielded a larger percentage of women screened in a shorter time frame compared to in-person recruitment. Furthermore, a large percentage of eligible women went on to enroll in the study. Study procedures and intervention content had to be adapted to reflect the remote delivery, but because the groundwork for the study was primarily technology based, it was a feasible and successful transition that took place over the course of 3 months. Our study team had to carefully consider potential issues related to participant safety, given that we no longer had in-person access to clinical care providers in the event of an emergency. This resulted in revisions to our procedures for how to respond to the disclosure of suicide risk and child abuse and neglect reporting. Our study assessments—both the original and modified versions—do not include questions explicitly asking about suicide risk. Rather, if a participant spontaneously shares a desire or plan to hurt themselves and/or suicide or self-harm is explicitly mentioned, we have in place an adapted protocol of action steps to remotely assess suicidality risk (ie, low, moderate, or high). For example, if a participant reports any suicidal intent, but indicates that she has no likelihood of acting on these thoughts in the near future, our staff provides her with resources (eg, National Suicide Prevention Lifeline and local resources, including social work) and offers to notify their primary care physician or mental health provider. If the participant discloses that they are somewhat or very likely to harm themselves, our research staff expresses concern and recommends that they speak to a professional as soon as possible. They also contact the principal investigator (PI) or clinical backup of the study who would plan to follow up with them by phone immediately. The research staff would provide numbers for local crisis and emergency mental health services within our health system that are offered. The PI would take appropriate action depending on the level of risk (eg, high risk would require a referral to the emergency department closest to the study participant). To date, we have not had a participant disclose suicidality.

### Conclusions

Despite study challenges related to the pandemic, including time and effort adapting to a remote protocol, remote recruitment and remote visits for this study were found to constitute a successful approach. We also recognize the potential advantages to participants in accessing our study remotely, including lessening the burden of time, costs, and inconvenience associated with traveling for in-person visits at the clinic [14]. It is important to note, however, that there are limitations to remote delivery of study visits, which may pose challenges for generalizing to other research studies or clinical settings that may not offer remote care. First, because study participation requires access to a telephone and a device with a reliable internet connection, it is possible that we could exclude low-income and marginalized women who might not have access to the required technology, and who are also the most vulnerable to negative health outcomes. Second, in our remote protocol, we are no longer able to collect biological (ie, hair and urine) samples as a secondary measure for drug use. Third, as this study is ongoing, our outcomes are pending. Notwithstanding these unknowns, just as COVID-19 has likely permanently changed the way we seek and utilize health care, it has also altered the way we conduct clinical behavioral research. In both instances, there are many benefits to embrace and maintain as we move forward.

## Abbreviations

HCEM: Health Check-Up for Expectant Moms
PI: principal investigator
STI: sexually transmitted infection
